# Publishing in the time of COVID-19

**DOI:** 10.7554/eLife.57162

**Published:** 2020-03-25

**Authors:** Michael B Eisen, Anna Akhmanova, Timothy E Behrens, Detlef Weigel

**Affiliations:** 1 eLifeCambridgeUnited Kingdom

**Keywords:** scientific publishing, peer review, preprints, early-career researchers, COVID-19

## Abstract

eLife is making changes to its policies on peer review in response to the impact of COVID-19 on the scientific community.

The SARS-CoV-2 virus is having a devastating impact as coronavirus disease 2019 (COVID-19) continues to spread in communities around the world. Many health-care professionals among us are battling on the front lines of this pandemic, seeing patients and helping to develop treatment and research protocols in real time. Scientists from diverse fields are redirecting their research to help develop diagnostics, therapies and vaccines. And all across the globe, labs are closed and people are working from home while worrying about their family, friends and colleagues, both near and far. 

In this time of crisis and uncertainty, we want to emphasize to everyone that nothing is more important than taking care of your health and sanity, and that of everyone around you. Publishing will not and should not be anybody’s first priority in the coming months.

However, it does seem that quite a few colleagues plan to take time away from their workplaces to write papers, and we will do everything we can to keep the journal running smoothly for them. We have surveyed our editors, and while some – especially clinicians and experts in virology and public health – will have to cut back on journal duties, most say they expect to be able to handle a roughly normal load of papers.

But even if people find the time to write papers about work they have already done, data they planned to collect may be in limbo, and it will be impossible for many to do additional experiments. And at a time when the speed of scientific progress is more important than ever, and people have a lot of other important things to attend to, the last thing we want to do is for publishing to contribute to delays.

We think it is essential that eLife changes how it functions in response to this new reality, so we are planning to take the following steps.

## 1. Curtail requests for additional experiments during revisions

eLife has always taken a strong stand against reviewers and editors imposing additional work on authors, and it has been our policy to restrict requests for new experiments or analyses to those that can reasonably be completed in two months (see elifesciences.org/about/peer-review). This policy was meant to strike a balance between the desire to let authors control their own science, and the reality that sometimes additional experiments make papers stronger.

But one result of this policy is that editors and reviewers naturally compare the submitted manuscript to an envisioned version of the paper that includes a few extra months’ work. This almost always makes the paper in hand appear wanting. However, given that many authors will be unable to do additional experiments for the foreseeable future, this dynamic must change.

We are therefore asking editors to accept without delay submitted manuscripts that in their judgment can stand as eLife papers, even if they feel that the manuscript would be stronger with additional data. In such cases, we will limit requests for revision to issues of clarity and presentation.

If the editors determine that the work as a whole belongs in eLife, but some conclusions require a modest amount of additional new data, we will ask for revisions that either alter the claims or make clear that the relevant conclusions require additional supporting data. If such revisions are made, the paper will be accepted for publication in eLife with the expectation that the authors will eventually carry out the additional experiments and report on how they affect the relevant conclusions either in a preprint on bioRxiv or medRxiv, or if appropriate, as a Research Advance in eLife, either of which would be linked to the original paper.

Papers which the editors judge to have potential, but where new data are needed to meet the standards of eLife, will be treated as normal revisions. We will, at the authors’ discretion, post the manuscript to bioRxiv along with our reviews and a formal declaration that the manuscript is ‘in revision at eLife’ (which the authors can cite on their CVs and in applications for jobs and grants).

We believe these changes will help authors whose research has been affected by the pandemic to publish their work without compromising eLife’s standards.

## 2. Suspend the two-month limit on revisions

In recognition of the fact that revisions may take longer than the two months we typically allow, until the research enterprise restarts in full, we will give authors as much time as they need to submit revised manuscripts. This applies to papers currently in revision and any submissions made during the pandemic.

## 3. Make the posting of preprints to bioRxiv or medRxiv the default for all eLife submissions

One of the most inspiring things about research in the age of COVID-19 has been the way that scientists studying the many manifestations of the virus and the pandemic have embraced preprint servers, and how this has greatly sped up the pace of research into understanding, preventing and treating the disease, and has also facilitated public understanding of what we are facing. eLife has always supported preprinting, and we believe that author-driven, immediate publishing is the future of research. We will continue to let authors decide whether preprinting is appropriate for their manuscript. But to emphasize how important we think speed is when conveying new research, we will now make posting to bioRxiv or medRxiv – either by the authors or the journal – the default for all eLife submissions. Authors will be able to opt out, but we will strongly encourage them not to.

Of course, posting to a preprint server is only part of the process: it is still essential (arguably even more so) that works get peer reviewed, and this is the main function of eLife and other journals. We have begun experimenting with a new system to attach eLife peer reviews to manuscripts on bioRxiv, and will roll this out more broadly as staffing and technology allow.

## 4. Extend our 'scoop protection' policy to cover competing work that is published on preprint servers prior to submission

It has always been eLife policy that the novelty of a finding is not diminished by related work published while a paper is under review at eLife (see ‘Beyond scoops to best practices’ and ‘FAQ on preprints and scoop protection’). With many authors posting preprints as a means to establish priority, to avoid people feeling pressure to post their papers prematurely, we wish to emphasize that our scoop protection policy applies to preprints as well as peer-reviewed journal articles. Specifically, if our editors consider that work has been done simultaneously by multiple groups, we will not consider any of the groups’ efforts to have been rendered less significant by the posting of work from another.

Note that this is not a change in our approach to submissions that report attempts to replicate previously published studies: we have always had a policy of publishing replications or failures to replicate that we regard as important, and we will continue to do so.

## 5. Mobilize early-career researchers

For the past few years eLife has been working with a global group of early-career researchers to increase their involvement in publishing. We carried out a successful trial of encouraging the use of early-career researchers as reviewers (see ‘Reflections on focused inclusion in reviews at eLife’), and now do this across the journal. We will now bring forward our plan to invite early-career researchers to be eLife reviewing editors, as an effort both to increase opportunities for them to gain editorial experience, and to diversity and expand our editorial board.

One last note. We ask for your patience on behalf of all the people who work incredibly hard to make eLife an amazing journal – our editors, reviewers and staff. Just like the rest of you, they have a lot going on and, while we hope to operate at peak efficiency, this may not be realistic for a while.

